# Data-driven segmentation of type 2 diabetes mellitus patients: an observational study on health care utilisation prior to an emergency department visit in Germany

**DOI:** 10.3389/fmed.2025.1509220

**Published:** 2025-05-16

**Authors:** Mirjam Rupprecht, Alessandro Campione, Yves Noel Wu, Antje Fischer-Rosinský, Anna Slagman, Dorothee Riedlinger, Martin Möckel, Thomas Keil, Lukas Reitzle, Cornelia Henschke

**Affiliations:** ^1^Department for Infectious Disease Epidemiology, Robert Koch Institute, Berlin, Germany; ^2^Department of Health Care Management, Berlin Centre for Health Economics Research, Technische Universität Berlin, Berlin, Germany; ^3^Emergency and Acute Medicine (CVK, CCM), Charité – Universitätsmedizin Berlin, Berlin, Germany; ^4^Institute of Social Medicine, Epidemiology and Health Economics, Charité – Universitätsmedizin Berlin, Berlin, Germany; ^5^Institute of Clinical Epidemiology and Biometry, University of Würzburg, Würzburg, Germany; ^6^State Institute of Health I, Bavarian Health and Food Safety Authority, Erlangen, Germany; ^7^Department of Epidemiology and Health Monitoring, Robert Koch Institute, Berlin, Germany; ^8^Faculty of Medicine, Institute of General Practice and Interprofessional Care, University Hospital Tübingen, Eberhard Karls Universität Tübingen, Tübingen, Germany

**Keywords:** type II diabetes mellitus, emergency department, health care utilisation, avoidable hospital admission, population segmentation, latent class analysis

## Abstract

**Background:**

Potentially avoidable hospital admissions (PAHs) due to type 2 diabetes mellitus (T2DM) occur more frequently in Germany than in the rest of Europe. Emergency departments (EDs) play an important role in understanding cross-sectoral health care utilisation resulting in inpatient admissions. Segmenting T2DM patients in homogenous groups according to their health care utilisation may help to understand the population’s needs and to allocate limited resources. The aim of this study was to describe ED use and subsequent inpatient admissions among T2DM patients, and to segment the study population into homogenous subgroups based on disease stage, health care utilisation and process quality of outpatient care prior to an ED visit.

**Methods:**

This study was conducted as part of the INDEED project, comprising data on 56,821 ED visits in 2016 attributable to 40,561 patients with T2DM from 13 German EDs, as well as statutory health insurance claims data from 2014 to 2016 retrospectively linked per patient. Descriptive analyses included patient characteristics, ED admission diagnoses and discharge diagnoses in the case of inpatient admission of T2DM patients to the ED. Latent class analysis was conducted to identify different subgroups of T2DM patients based on disease stage, number of physician contacts and medical examinations prior to the ED visit.

**Results:**

Almost half of the study population had severe comorbidities (44.3%). In addition to T2DM, multiple cardiovascular diagnoses were among the most frequently documented admission and discharge diagnoses. The proportion of hospitalised ED visits for T2DM patients was higher (59%) than that for the INDEED population (42.8%). We identified three latent classes that were characterised as *“early disease stage and high utilisation*” (36.5% of the study population), “*progressing disease stage and low utilisation”* (26.1%) and *“progressed disease stage and high utilisation”* (37.4%).

**Conclusion:**

A substantial share of T2DM patients had not received disease monitoring according to guideline recommendations prior to ED presentation. Improving guideline-adherence in the outpatient sector could help reduce potentially avoidable ED visits. Effective interventions that aim at improving continuity and quality of care as well as reducing the share of PAH need to be identified and evaluated per identified class.

## 1 Introduction

Type 2 diabetes mellitus (T2DM) is a chronic metabolic disease characterised by elevated blood glucose levels. In Germany, 8.6% (95% CI: 7.4–10.0%) of women and 9.9% (95% CI: 8.6–11.3%) of men aged 18 to 79 are affected by known and unknown diabetes mellitus (DM) (1). The prevalence of known DM in Germany (8.4%; 95% CI: 7.8–8.9%) is thus higher than the EU average of 7.4% (95% CI: 7.3–7.6%) (2, 3). In the adult population, T2DM is the most common type of diabetes mellitus, accounting for more than 90.0% of all diagnosed cases (4, 5). Despite existing treatment guidelines and the high potential for preventing disease progression, DM frequently leads to comorbidities, including diseases of the cardiovascular system, kidney failure, diseases of the eye and amputations of the lower extremities (4). Due to the high burden of disease and the high economic burden on healthcare systems, DM constitutes a substantial challenge for public health (6).

DM is classified as an ambulatory care sensitive condition (ACSC) (7, 8), i.e., DM-related inpatient admissions can be avoided with adequate, guideline-based outpatient treatment (9). Potentially avoidable hospital admissions (PAHs) due to T2DM are defined as a key indicator to measure quality of care in the outpatient sector by the Organisation for Economic Co-operation and Development (OECD) and are monitored as part of the German Diabetes Surveillance (2, 62). With an inpatient admission rate of 206 per 100,000 people, Germany has high levels of PAH due to DM compared to the EU average admission rate of 139 per 100,000 people (2). In addition, DM is also a risk factor for other diseases leading to PAH: In a French cohort of patients with DM, congestive heart failure and chronic obstructive pulmonary disease (COPD) were the two most common causes of inpatient treatment due to an ACSC, while short-term complications due to DM ranked third (10). A Portuguese study on the impact of DM on multiple inpatient admissions showed that inpatients diagnosed with DM had a longer average length of stay and higher costs (11). In Germany, the prevalence of DM in inpatient cohorts is twice as high as in the general population, and inpatient mortality is increased by up to 30% in individuals with T2DM compared to non-diabetics (12).

Continuous, coordinated care across sectors is crucial for preventing PAHs (9). Mapping and improving cross-sectoral care play an important role in the highly fragmented German healthcare system. The financial and organisational separation of the outpatient and inpatient sectors in Germany impairs the coordination and quality of health care services (13). Emergency departments (EDs) form a special interface between the outpatient and inpatient sectors, as they contain components of both sectors (14). While services for patients not admitted as inpatients are billed on an outpatient basis through the associations of statutory health insurance (SHI) physicians organised at the federal state level, services for patients admitted as inpatients are billed directly by health insurance funds (15). With 48.8%, the inpatient admission rate out of all ED visits is significantly higher in Germany than in other European countries (16). EDs thus play a relevant role in understanding cross-sectoral patterns of health care utilisation resulting in inpatient admission. To date, little is known about the cross-sectoral health care utilisation of T2DM patients attending EDs.

To improve cross-sectoral coordination of care and enhance patient management of T2DM patients, it is important to understand health care utilisation resulting in ED visits and inpatient admissions (8). Segmenting the population into homogenous groups according to their health care utilisation contributes to a better understanding of the population’s demand and facilitates the effective allocation of limited resources from a population health perspective (17, 18). Usually, in population segmentation methods, a distinction is made between data-driven and expert-driven approaches. In expert-driven approaches, segments of a population are distinguished by a panel of experts or health care practitioners using predefined criteria established through a literature review or a consensus-building process. Data-driven approaches use statistical analyses on extensive datasets to recognise underlying patterns of factors influencing predefined outcomes (18). Regarding T2DM patients, the most studied objectives to date are health grouping, assessment of diabetes-related complications, and non-diabetic metabolic complications. Segmentation variables at the individual level mainly included sociodemographic, DM-related, and non-DM medical-related variables. Variables relating to the healthcare system utilisation are used less frequently (17). To date, no data-driven segmentation analyses have been conducted on the outpatient health care utilisation of T2DM patients prior to an ED visit.

The overall aim of this analysis was to contribute to a better understanding of cross-sectoral patterns of health care utilisation among T2DM patients prior to ED visits with and without subsequent inpatient admissions via ED in Germany. Specifically, the study aimed to (1) describe their outpatient health care utilisation in the year prior to an ED visit, (2) describe their diagnoses in the ED as well as main hospital diagnoses in case of inpatient admission, and (3) identify homogenous subgroups of T2DM patients regarding disease stage, outpatient health care utilisation and process quality of outpatient health care prior to an ED visit.

## 2 Methods

### 2.1 Data source

This study was conducted as part of the explorative multicentre project INDEED (INDEED: Utilisation and cross-sectoral patterns of care of patients in emergency departments in Germany). The overall aim of the INDEED project was to illustrate ED use and to explore cross-sectoral patterns of care for patients admitted to EDs (14, 19). The INDEED dataset used for this study consisted of (1) ED data of SHI insured patients collected from 16 German EDs in 2016 and (2) outpatient claims data from the years 2014–2016. Data were collected and linked for each of the ED patients retrospectively (14). The data set is unique for Germany. A more recent, comparable database with linked ED and outpatient data is not available due to the established data structures and data protection regulations in Germany. Over the last two decades the number of ED cases increased constantly, although this changed during the covid-19 pandemic (20). Nevertheless, the data set is still suitable for investigating cross-sectoral patterns of health care utilisation in Germany.

The ED data included information on patient demographics, ED treatment and ED diagnoses. The ED data were supplemented with the main hospital diagnosis in the case of inpatient admission following the ED visit. Diagnoses were not available for every ED visit or inpatient admission. Three of the 16 EDs did not provide any ED diagnoses and were therefore excluded from further analyses. The outpatient data included information on patient demographics and insurance status, as well as outpatient diagnoses coded according to ICD-10-GM (International Classification of Diseases and Related Health Problems, 10^th^ revision, German Modification) (21), medical procedures coded according to standardised outpatient billing codes (22), and drug prescription data coded according to ATC-codes (Anatomical Therapeutic Chemical Classification) (23). Information on participation in a disease management programme (DMP) appeared to be incomplete, as the numbers of documented participants for all DMPs were significantly lower than the actual numbers recorded by statutory health insurances. It was not possible to ascertain whether this discrepancy was a data artefact or an actual deviation in the study population from the total population, based on the available data. Outpatient data were transmitted from eight associations of SHI physicians in the federal states in which the participating EDs were located (24). One of the eight participating associations of SHI physicians was unable to transmit drug prescription data. Comprehensive information on the participating EDs and associations of SHI physicians as well as the process of data linkage is described elsewhere (19).

The dataset is based on a total of 454,747 ED visits in 2016, attributable to 353,926 patients, who were at least 18 years old in 2014. Furthermore, the dataset only contained ED visits that were billed via the SHI. In 2020, 87% of the German population were covered by statutory health insurance (13). Privately insured patients and visits billed via statutory accident insurance were not included in the dataset (25).

### 2.2 Study population

#### 2.2.1 Definition

**Figure 1:**
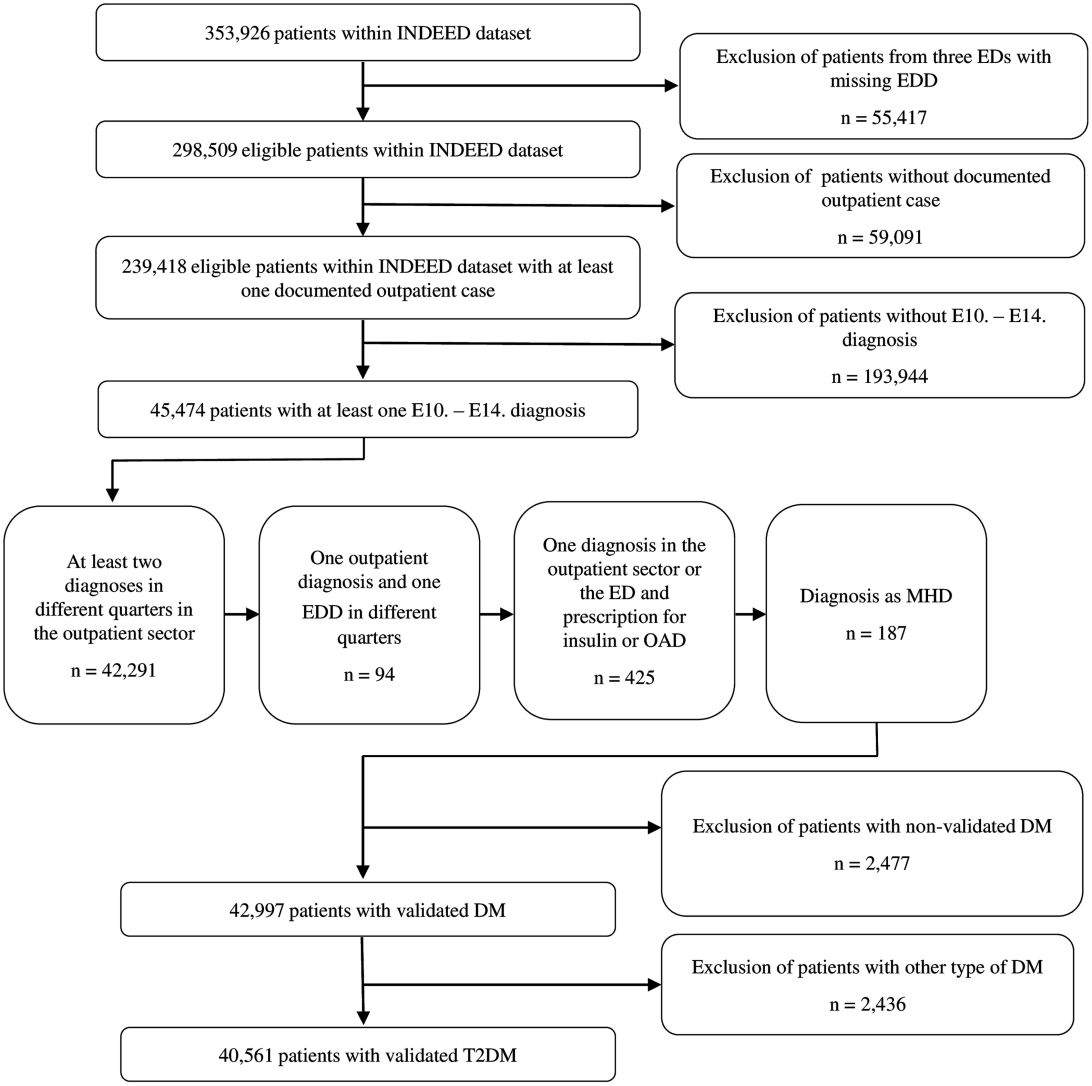
Flowchart of patient selection of all patients with validated type 2 diabetes mellitus within the INDEED dataset. DM, Diabetes mellitus; ED, Emergency department; EDD, Emergency department diagnosis; MHD, Main hospital diagnosis; OAD, Oral antidiabetic drug; T2DM, Type 2 diabetes mellitus.

The study population included patients with a validated T2DM diagnosis for which at least one case was documented in the outpatient data. The presence of DM was defined via the ICD-10-GM-Codes (E10.- up to E14.-) (21). In addition, drug prescriptions of insulin and oral antidiabetic drugs (OADs) as well as participation in a DMP (if available) were included for validation of diagnosis and to distinguish between different types of DM. Prescriptions for insulin or OAD were identified via ATC-codes (23). Table 1 provides an overview of the codes considered. Figure 1 illustrates the process of extraction and validation of the study population as outlined below.

**Table 1:** Overview of the codes used for study population definition.

Inclusion criteria	Code	Description
Diagnoses according to ICD-10-GM (21)	E10.-	Type 1 diabetes mellitus
	E11.-	Type 2 diabetes mellitus
	E12.-	Diabetes mellitus associated with malnutrition
	E13.-	Other diabetes mellitus with further details
	E14.-	Other diabetes mellitus without further details
Drug prescriptions according to ATC (23)	A10A	Insulin and analogues
	A10B	Antidiabetics, excl. Insulin
Participation in disease management programme (DMP), if available	T1DMP	Participation in DMP for type 1 diabetes mellitus
	T2DMP	Participation in DMP for type 2 diabetes mellitus

#### 2.2.2 Extraction and validation

The extraction of the study population was performed in three steps in accordance with procedures recommended by the Robert Koch Institute (RKI) and the German Institute for Quality and Efficiency in Health Care (IQWiG) (4, 26). (I) All patients with at least one ICD-10-GM code for diabetes mellitus were identified within the dataset. Outpatient diagnoses two years prior to the ED visit, as well as ED diagnoses and the main hospital diagnosis were considered. The period “two years prior to the ED visit” comprised eight quarters prior to the quarter in 2016 in which the ED visit took place for each patient. In the case of multiple ED visits per patient in 2016, the first visit was used as the calculation basis. Only outpatient diagnoses with the addition of “confirmed” or “condition after” were considered to ensure diagnosis validity (5, 26). Inpatient diagnoses are considered valid in the German healthcare system due to standardised coding guidelines with billing relevance (27). (II) The presence of at least one of the following conditions was used to validate the DM diagnosis: (1) DM was coded at least two quarters in the outpatient data two years prior to the ED visit; (2) DM was coded once in the outpatient data two years prior to the ED visit and once as the ED diagnosis in 2016; (3) DM was diagnosed once in the outpatient data two years prior to the ED visit or as the ED diagnosis in 2016, and there was at least one prescription for insulin or OAD in the outpatient data two years prior to the ED visit; or (4) DM was coded as main hospital diagnosis in case of inpatient admission following the ED visit. The conditions were applied one after another, i.e., if condition one was fulfilled, the following conditions were not applied. Conditions one to three were defined in reference to the well-validated M2Q criterion, which describes the repeated documentation of a diagnosis over the course of a year, usually in two or more different quarters (28, 29). In deviation from the usual definition of the criterion, the presence of at least two quarters with a documented DM diagnosis or a DM-relevant drug prescription within two years, not just one year, was defined as sufficient for diagnosis validation in order to identify all cases. The quarters with DM diagnosis or prescription did not have to be consecutive, and diagnosis and prescription of antidiabetic drugs did not have to be in the same quarter. (III) Finally, the study population was further narrowed to persons with T2DM only, based on an allocation algorithm considering the presence of different combinations of E10.- to E14.- diagnoses, insulin or OAD prescriptions, and participation in a DM-specific DMP (if available, Appendix 1).

### 2.3 Analysis

Descriptive analyses were performed to summarise patient characteristics, health care utilisation prior to the ED visit, as well as ED diagnoses and main hospital diagnoses. Latent class analysis (LCA) was applied for population segmentation. LCA is a probabilistic, unsupervised clustering method used to determine whether unobserved homogenous subgroups (*classes*) exist within populations based on measured indicator variables. In contrast to other clustering methods such as k-means, individuals are not assigned to a cluster by distance measures. Instead, maximum likelihood estimations, based on finite mixture modelling of assumed underlying latent classes are used to calculate probabilities of individuals belonging to all latent classes in the model (*posterior probability*). An LCA is appropriate for large study samples and categorical dependent variables (*indicator variables*) (30). It is particularly applicable for identifying subgroups that could benefit from similar interventions (31). One of the main prerequisites of LCA is the assumption of *“local independence,”* i.e., indicator variables are independent of one another. Violations of this assumption can lead to misclassification due to data redundancy and result in low accuracy of fit statistics and overestimation of the true number of classes (30).

#### 2.3.1 Indicator variables

Table 2 describes the selected indicator variables. Age and sex represented patient demographics. The number of documented DM-related complication groups and DM-specific medication indicated the disease stage of T2DM (63). The following variables represent quality markers for T2DM outpatient care. The number of contacts with physicians in the outpatient sector characterises different levels of health care utilisation (32). Accessibility of physicians in the outpatient sector was inversely associated with PAH, although conflicting results exist (33). The differentiation of the medical specialty groups was based on the levels of the German planning scheme for the regional distribution of SHI physicians and was further restricted to disciplines relevant for T2DM patients (Appendix 3) (34). As flat rates are billed on a quarterly basis in outpatient physician care, no statement can be made regarding the actual number of physician contacts per quarter (29). All health care utilisation variables were therefore calculated on a quarterly basis. HbA1c (glycated haemoglobin) and microalbuminuria measurements, as well as ophthalmological fundus examination, are recommended as indicators for outpatient process quality in the treatment of DM (35, 36). In addition, creatinine measurement was defined as a mandatory annual measurement in the German guidelines for DMP for T2DM (37). Since microalbuminuria measurement is particularly relevant for the early detection of diabetic nephropathy and since creatinine is used as an indicator of disease progression, both indicators were considered (38). According to the German guidelines for DMP for T2DM, the HbA1c should be checked quarterly, but at least twice a year. Microalbuminuria and creatinine should be checked annually. The recommended frequency for ophthalmological fundus examination was reduced in 2015 from annually to at least biennially for people without a known risk for retinopathy (37). Regular measurements of HbA1c and microalbuminuria were shown to be negatively associated with inpatient admissions (39).

**Table 2:** Description of variables used for population segmentation.

Variable	Description
Patient characteristics and stage of disease	
Sex	Sex is classified as male (1) or female (2).
Age	Age in years at the time of the ED visit in 2016. In the case of multiple ED visits in 2016 with differing ages the mean value was calculated. Age was categorised in four groups: 0–55 (1), 56–70 (2), 71–85 (3), ≥ 86 (4).
DM-related complication groups	The number of documented DM-related complication groups one year prior to the ED visit was defined based on the fourth digit of ICD-codes starting with E10 - E14 and further DM-specific codes. The following groups of complications were defined: metabolic system, eye, kidney, neuro-vascular system, multiple complications.^1^ The number of documented complication groups was categorised in three groups: 0 (0), 1–2 (1), ≥ 3 (2).
T2DM specific medication	The use of DM-specific medication was estimated on drug prescriptions one year prior to the ED visit. Medication was categorised in four groups: no DM-specific medication (0), only OAD (1), only insulin (2), OAD and insulin (3).
Health care utilisation	
General practitioner visits	The number of quarters in which any billing code was claimed by a general practitioner in the year prior to the ED visit.^2^ The number of quarters with contact was categorised in three groups: 0 (0), 1–2 (1), 3–4 (2).
General specialist visits	The number of quarters in which any billing code was claimed by a general specialist in the year prior to the ED visit.^2^ The number of quarters with contact was categorised in three groups: 0 (0), 1–2 (1), 3–4 (2).
Specialised specialist visits	The number of quarters in which any billing code was claimed by a specialised specialist in the year prior to the ED visit.^2^ The number of quarters with contact was categorised in three groups: 0 (0), 1–2 (1), 3–4 (2).
Process quality of outpatient care	
HbA1c measurement	The number of quarters in which the billing code for HbA1c measurement was claimed in the year prior to the ED visit. The number of quarters with measurement was categorised in three groups: 0 (0), 1–2 (1), 3–4 (2).
Microalbuminuria measurement	The number of quarters in which the billing code for microalbuminuria measurement was claimed in the year prior to the ED visit. The number of quarters with measurement was categorised in three groups: 0 (0), 1–2 (1), 3–4 (2).
Creatinine measurement	The number of quarters in which one of the billing codes for creatinine measurement was claimed in the year prior to the ED visit. The number of quarters with measurement was categorised in three groups: 0 (0), 1–2 (1), 3–4 (2).
Ophthalmological fundus examination	The number of quarters in which one of the billing codes for fundus examination or fluorescence angiographic examination was claimed in the year prior to the ED visit. The number of quarters with measurement was categorised in three groups: 0 (0), 1–2 (1), 3–4 (2).

ED, Emergency department; HbA1c, Glycated haemoglobin; OAD, Oral antidiabetic drugs; T2DM, Type 2 diabetes mellitus. ^1^Included codes are listed in Appendix 2. Classification of complication groups based on (47). ^2^Included disciplines are listed in Appendix 3. Billing codes regarding administrative activities or laboratory tests were excluded, as these billing codes do not require direct contact with a physician.

The indicators were calculated per patient. The period “one year prior to the ED visit” comprised the four quarters prior to the quarter in 2016 in which the ED visit took place. In the case of multiple ED visits per patient, the first visit was used as the calculation basis. Diagnoses, procedures, or medications were defined as not existing or not performed per patient as soon as the patient was present at least once in the outpatient data, but the corresponding billing code was not used in the defined period. Variable values were collapsed to three to four categories per indicator to ensure interpretability of the classes (31).

#### 2.3.2 Data setup

Patterns of missing data are illustrated. Random forest imputation was conducted with ten iterations to impute missing data (40). Random forest imputation is a fully conditional, non-parametric imputation method that makes no assumptions about normality, linearity of the relation between variables, homoscedasticity, or independence (41). After imputation, collinearity between the indicator variables was checked to validate the assumption of local independence, whereas correlation coefficients under 0.50 were considered (30).

#### 2.3.3 Model selection and validation

The selection of the number of clusters is a challenge in all segmentation methods, since universally valid latent clusters do not exist, and definitions of clusters are strongly influenced by the underlying research objective (40). To determine the number of classes, the use of various indices is recommended (42). Regarding information criteria indicating model fit, the use of the Bayesian information criterion (BIC) is recommended for large samples, with smaller values indicating better model fit (42). For very large samples, it is recommended to apply the elbow heuristic to the BIC, i.e., to find the inflection point in the BIC plot from which the decrease in values is less steep (30). To clearly identify distinct clusters, entropy-based criteria were used, with the integrated completed likelihood (ICL) proving more accurate than scaled relative entropy (42). In addition, the average silhouette width (ASW) can be used to check for within-cluster dissimilarities and cluster separation, with higher values indicating less dissimilarity (40). The ASW is usually applied in distance-based clustering methods. However, simulations have shown that model performance regarding the ASW does not differ between distance-based and probabilistic models (43). Theoretical interpretability should be considered at least as important as statistical fit values (30, 31, 42).

The selection of the final class solution was conducted in accordance with the extended selection strategy proposed by Lezhnina and Kismihók (40). The approach does not rely solely on the model fit indicated by information criteria but also considers cluster separation and partition stability. To determine the number of classes, LCA models were fitted for 1- to 10-class solutions. The selection of class solutions was then conducted under consideration of the BIC elbow heuristic, maximum ASW and minimum ICL to handle the trade-off between cluster separation and model fit (40). The smallest identified latent class in the chosen model was checked to comprise at least 5% of the study sample (31).

For internal validation, partition stability was checked via adjusted Rand index (ARI) and Jaccard index, whose values can range from zero to one and values closer to one indicate better stability (40). Furthermore, an average latent class posterior probability of at least 0.80 per class was used to assess the classification accuracy of the chosen model (31). Model selection was performed only on a proportion of the total sample, using a split ratio of 0.7 to separate training and validation data, to avoid overfitting and improve out-of-sample fit. For validation, the partition stability and classification accuracy of the chosen model were confirmed in the validation data.

#### 2.3.4 Model interpretation

The characteristics of the classes per indicator variable as well as differences in class patterns were analyzed. The ten most frequently coded ED diagnoses and main hospital diagnoses per class were compared. Patient characteristics, recurrent ED visits and inpatient admissions per class were described. Recurrent ED visits and inpatient admissions were coded dichotomously. Recurrent ED visits were classified as present if there was more than one ED visit documented per patient in the participating EDs in 2016. Inpatient admission was classified as present as soon as at least one inpatient admission was documented per patient. Univariate and age-adjusted logistic regressions were performed to detect significant differences in the odds of recurrent ED visits or inpatient admissions between the classes, using the age categories described in Table 2 for age adjustment. Age was already defined as one of the discriminatory variables in the LCA, but showed little discriminatory power within the LCA (see Appendix 8). As age is known to influences both health care utilisation and disease progression, an additional model adjusted for age was created for comparison.

All steps were performed using the statistics program R version 4.3.2. The *comorbidity* index package was used to estimate the Charlson comorbidity index (44). The *missforest* package was used for the imputation of missing data (41, 45). The model selection function provided by Lezhnina and Kismihók is based on the VarSelClust function from the *VarSelLCM* package (40, 46).

### 2.4 Ethics and data protection

The study was approved by the ethics committee of the Charité – Universitätsmedizin Berlin (application number EA4/086/17). The data protection concept was approved by the data protection working group of the Technology and Methods Platform for Interconnected Medical Research e.V. (TMF) and the data protection officer of Charité – Universitätsmedizin Berlin (registration number 565/17/ST3). INDEED is registered in the German clinical trials registry (registration number DRKS00022969) (14).

## 3 Results

### 3.1 Study population

As shown in Figure 1, out of 239,418 eligible patients within the INDEED dataset, 40,561 patients fulfilled the inclusion criterion of having a validated diagnosis of T2DM and at least one documented outpatient case. The median age of the study population was 75 years. Almost half of the study population had severe comorbidities with CCI scores higher than four (44.3%). In 2016, a total of 56,821 visits to the included EDs were recorded. Of the 40,561 patients with T2DM, 30,844 patients had only one recorded visit, while 9,717 had two or more. Overall, 59.0% of all visits led to inpatient admissions. At least one inpatient admission was recorded for 65.1% all patients with T2DM.

Table 3 provides additional information on the study population, categorised into patients with at least one inpatient admission and those without. This distinction is intended to show whether differences in socio-demographic factors, disease severity and the extent of outpatient care prior to the ED visit influence the need for inpatient admission.

**Table 3:** Summary of study population characteristics.

Patient characteristics	Study population *n* = 40,561 in % (*n*)	Patients with at least one inpatient admission *n* = 26,390 in % (*n*)	Patients without inpatient admission *n* = 14,171 in % (*n*)
Age in years			
20–55	11.5 (4,674)	7.8 (2,051)	18.5 (2,623)
56–70	26.8 (10,889)	25.2 (6,658)	29.9 (4,231)
71–85	48.7 (19,757)	52.5 (13,848)	41.7 (5,909)
≥ 86 years	12.9 (5,241)	14.5 (3,833)	9.9 (1,408)
Median (IQR)	75 (64, 81)	76 (67, 82)	71 (59, 79)
Sex			
Male	50.7 (20,563)	51.9 (13,704)	48.4 (6,859)
Female	49.3 (19,998)	48.1 (12,686)	51.6 (7,312)
Insurance status			
Member	23.0 (9,345)	18.8 (4,951)	31.0 (4,394)
Family insured	2.6 (1,071)	2.1 (542)	3.7 (529)
Retiree	74.3 (30,145)	79.2 (20,897)	65.3 (9,248)
Charlson comorbidity index^1^			
CCI Score 0	0.9 (379)	1.0 (255)	0.9 (124)
CCI Score 1–2	25.6 (10,369)	21.6 (5,708)	32.9 (4,661)
CCI Score 3–4	29.2 (11,852)	28.5 (7,518)	30.6 (4,334)
CCI Score ≥ 5	44.3 (17,959)	48.9 (12,908)	35.6 (5,051)
No outpatient diagnoses documented	<0.1 (2)	<0.1 (1)	<0.1 (1)
Median (IQR)	4 (2, 6)	4 (3, 6)	3 (2, 5)
Presence of DM-related complications^2^			
0 complication groups	51.9 (21,055)	49.3 (13,008)	56.8 (8,047)
1–2 complication groups	44.3 (17,976)	46.5 (12,261)	40.3 (5,715)
≥ 3 complications groups	3.8 (1,528)	4.2 (1,120)	2.9 (408)
No outpatient diagnoses documented	<0.1 (2)	<0.1 (1)	<0.1 (1)
Frequency of ED presentation in 2016^3^			
1–2 presentations/year	91.9 (37,288)	89.0 (23,495)	97.3 (13,793)
3–9 presentations/year	8.0 (3,226)	10.8 (2,854)	2.6 (372)
≥ 10 presentations/year	0.1 (47)	0.2 (41)	<0.1 (6)

CCI, Charlson comorbidity index; IQR, Interquartile range; ED, Emergency department; DM, Diabetes mellitus. ^1^According to Appendix 4, based on (44, 60); calculation basis: outpatient data one year prior to ED visit. ^2^According to Appendix 2; based on (47); calculation basis: outpatient data one year prior to ED visit. ^3^Categorisation according to (61); regarding visits in included EDs.

### 3.2 Health care utilisation prior to the ED visit

Table 4 provides information on medical outpatient health care utilisation in the year prior to the ED visit. Although 98.5% of patients had contact with a GP, 17.8% of patients did not have their HbA1c measured during the same period.

**Table 4:** Outpatient health care utilisation one year prior to the index emergency department visit in 2016.

Patient-based characteristics	Study population *n* = 40,561 in % (*n*)	Patients with at least one inpatient admission *n* = 26,390 in % (*n*)	Patients without inpatient admission *n* = 14,171 in % (*n*)
Medication for DM			
No DM-specific medication	29.6 (12,025)	29.2 (7,715)	30.4 (4,310)
OAD only	26.4 (10,710)	26.2 (6,922)	26.7 (3,788)
Insulin only	10.2 (4,151)	12.2 (3,223)	6.5 (928)
Insulin and OAD	13.3 (5,392)	14.6 (3,855)	10.8 (1,537)
No drug prescription documented	20.4 (8,283)	17.7 (4,675)	25.5 (3,608)
Number of quarters with documented			
Contact to a general practitioner			
0 quarters/ year	1.7 (701)	1.9 (513)	1.3 (188)
1–2 quarters/ year	3.6 (1,473)	3.2 (843)	4.4 (630)
3–4 quarters/ year	94.6 (38,387)	94.9 (25,034)	94.2 (13,353)
Contact to a general specialist			
0 quarters/ year	17.7 (7,185)	18.9 (4,986)	15.5 (2,199)
1–2 quarters/ year	28.8 (11,671)	28.9 (7,620)	28.6 (4,051)
3–4 quarters/ year	53.5 (21,705)	52.2 (13,784)	55.9 (7,921)
Contact to a specialised specialist			
0 quarters/ year	42.9 (17,387)	43.4 (11,493)	41.6 (5,894)
1–2 quarters/ year	38.9 (15,761)	37.8 (9,965)	40.9 (5,796)
3–4 quarters/ year	18.3 (7,413)	18.7 (4,932)	17.5 (2,481)
HbA1c measurement			
0 quarters/ year	17.8 (7,231)	18.1 (4,778)	17.3 (2,453)
1–2 quarters/ year	33.3 (13,489)	32.6 (8,616)	34.4 (4,873)
3–4 quarters/ year	48.9 (19,841)	49.2 (12,996)	48.3 (6,845)
Microalbuminuria measurement			
0 quarters/ year	84.3 (34,185)	84.3 (22,237)	84.3 (11,948)
1–2 quarters/ year	13.8 (5,607)	13.9 (3,677)	13.6 (1,930)
3–4 quarters/ year	1.9 (769)	1.8 (476)	2.1 (293)
Creatinine measurement			
0 quarters/ year	12.8 (5,174)	13.9 (3,419)	12.4 (1,755)
1–2 quarters/ year	42.4 (17,196)	41.1 (10,844)	44.8 (6,352)
3–4 quarters/ year	44.8 (18,191)	45.9 (12,127)	42.8 (6,064)
Ophthalmological fundus examination			
0 quarters/ year	67.0 (27,162)	68.0 (17,952)	65.0 (9,210)
1–2 quarters/ year	30.0 (12,174)	29.0 (7,657)	31.9 (4,517)
3–4 quarters/ year	3.0 (1,225)	3.0 (781)	3.1 (444)

ED, Emergency department; DM, Diabetes mellitus, HbA1c, Glycated haemoglobin; OAD, Oral antidiabetic drug.

### 3.3 Emergency department diagnoses and inpatient admissions

For 7,414 of the 56,821 ED visits no ED diagnoses were transmitted, affecting 6,715 patients. Among the remaining 49,407 ED visits, 110,448 diagnoses were coded, with an average of 2.4 diagnoses per ED visit. One of the 13 EDs was notably above average with 9.9 documented diagnoses per ED visit and was excluded from the following analysis of the top ten most frequently coded ED diagnoses (responding to 3,266 excluded patients accounting for 4,373 ED visits). Table 5 represents the ten most frequent ICD-codes documented in the remaining twelve EDs, considering the first three digits of the codes. It should be noted that diagnoses per patient may be coded more than once if they presented repeatedly at the ED in 2016.

**Table 5:** Top ten coded three-digit ICD-10-codes in the emergency department.

ICD-code	ICD-code description	Number of ICD-codes documented *n* = 67,196 in % (*n*)
I10	Essential (primary) hypertension	2.5 (1,679)
E11	Type 2 diabetes mellitus	2.1 (1,398)
M54	Back pain	1.7 (1,172)
S00	Superficial injury to the head	1.7 (1,138)
I63	Cerebral infarction	1.7 (1,115)
R10	Abdominal and pelvic pain	1.7 (1,112)
J18	Pneumonia, pathogen not specified	1.6 (1,103)
I50	Congestive heart failure	1.6 (1,046)
R07	Throat and chest pain	1.5 (1,009)
I48	Atrial fibrillation and flutter	1.5 (978)
	Total share of the 10 most frequent codes	17.6%

For T2DM patients, who were admitted to the hospital, the inpatient mortality rate was 6.6%. Table 6 represents the ten most frequent ICD-codes documented as main hospital diagnosis in case of inpatient admission. Details on the coded T2DM diagnoses in the ED and as main hospital diagnoses in case of inpatient admission can be found in Appendix 5.

**Table 6:** Top ten coded three-digit ICD-10-codes as main hospital diagnosis in case of inpatient admission.

ICD-code	ICD-code description	Number of ICD-codes documented *n* = 33,224 in % (*n*)
I63	Cerebral infarction	6.1 (2,032)
I50	Congestive heart failure	5.4 (1,784)
E11	Type 2 diabetes mellitus	3.1 (1,043)
S06	Intracranial injury	3.0 (1,004)
I21	Acute myocardial infarction	2.9 (957)
A41	Other sepsis	2.8 (943)
J18	Pneumonia, pathogen not specified	2.1 (714)
G45	Cerebral transient ischaemia and related syndromes	2.1 (706)
N17	Acute renal failure	2.1 (687)
J44	Other chronic obstructive pulmonary disease	2.0 (663)
	Total share of the 10 most frequent codes	31.6%

### 3.4 Population segmentation

#### 3.4.1 Data setup

At 20.4%, “medication” had the highest proportion of missing data, whereas all other variables showed proportions of missing values of less than 1 % (Appendix 6). More than 99.0% of the missing medication values occurred in patients from a particular association of SHI physicians, which did not provide drug prescription data. Random forest imputation was conducted. Local independence was checked via a correlation matrix (Appendix 7). A correlation of 0.63 was found between the indicators “HbA1c” and “creatinine measurement.” Therefore, “Creatinine measurement” was excluded from the LCA to avoid bias and misclassification and to reduce data redundancy (30).

#### 3.4.2 Model selection

**Figure 2:**
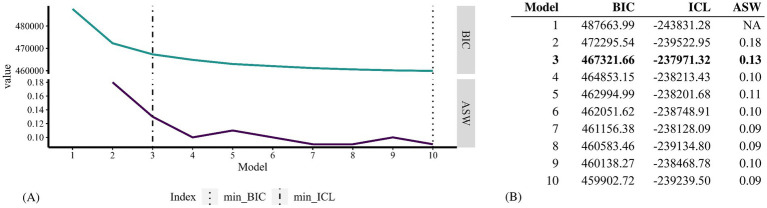
Visualisation of 1- to 10-class solutions **(A)** and cluster selection results **(B)** assessed for the latent class analysis. The model name shown on the x-axis indicates the number of clusters tested for the respective model.

By applying the combination of the BIC elbow criterion and the maximum ASW indicating model fit as well as the minimum ICL to assess cluster separation, the three-class solution was selected as the final model. This is illustrated in Figure 2, which shows the ASW and BIC values for each of the 1- to 10-class solutions along the x-axis, with the minimum ICL indicated by the dash-dot vertical line.

The “number of quarters with contact with a general specialist” showed the highest discriminatory power in the three-class solution, followed by “medication” (Appendix 8). The partition stability was stable, with an ARI of 0.92 and a Jaccard index of 0.91. At exactly 0.80 for class one, the smallest average latent class posterior probability reached the target value of 0.80 (Appendix 9). Partition stability in the validation dataset was at 0.89 (ARI) and 0.87 (Jaccard). With the smallest average latent class posterior probability at 0.80, the classification accuracy was at the targeted value in the validation dataset as well.

#### 3.4.3 Model interpretation

**Figure 3:**
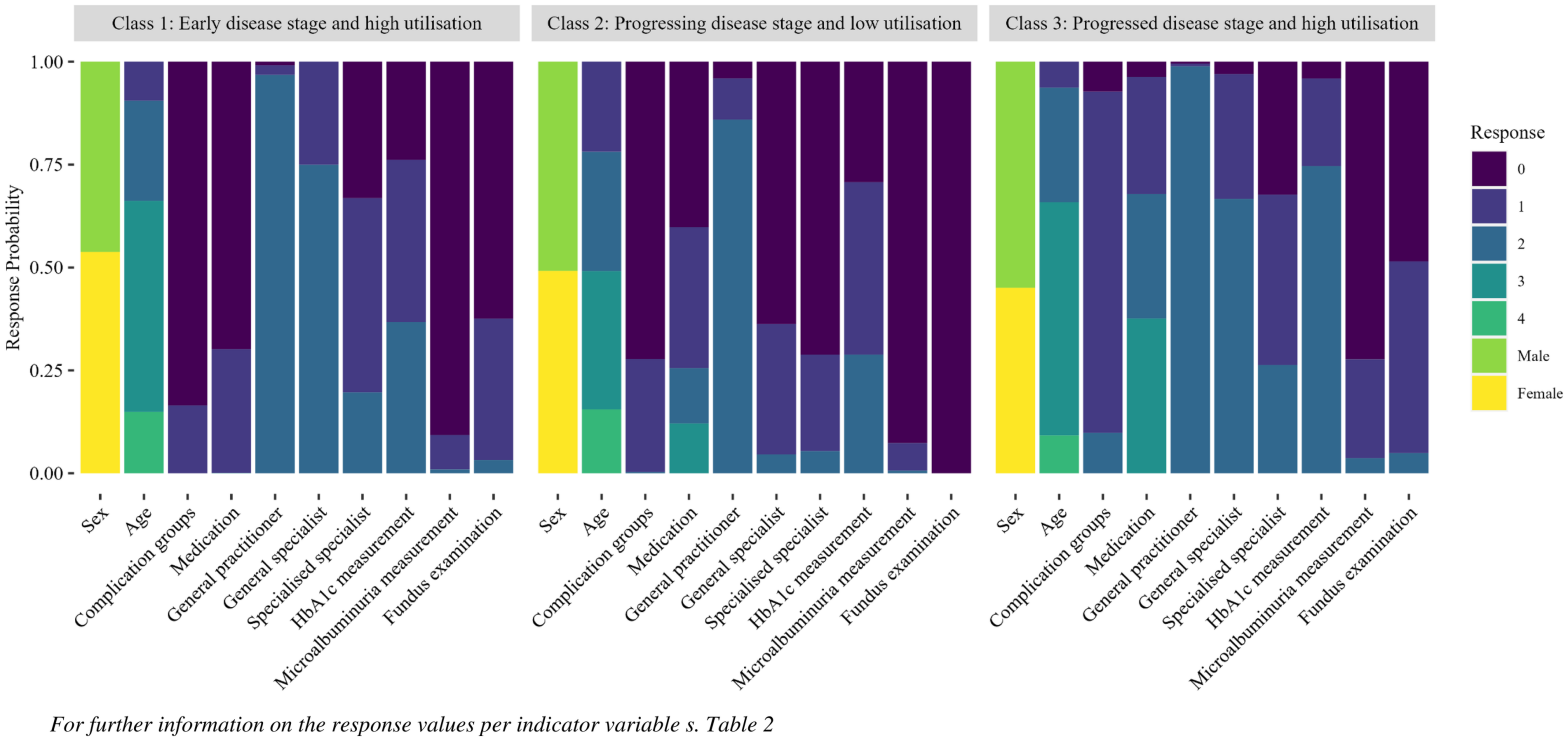
Response probability per indicator per class identified with the latent class analysis.

After model selection and validation, the model was applied to all 40,561 patients. The response distributions of the three identified classes per indicator variable are shown in Figure 3.

The first class was named *“early disease stage and high utilisation”* and represented 36.5% of the study population. Class one was characterised by low average values of indicators reflecting disease progression, with high health care utilisation. The second class, *“progressive disease stage and low utilisation,”* containing 26.1% of the study population, showed evidence of a more advanced stage of T2DM than class one, with higher values for “medication” in particular. However, compared to classes one and three, class two showed a lower utilisation of specialised care and less frequent measurement of all indicators reflecting process quality of care. The third class *“progressed disease stage and high utilisation,”* had the highest values of disease progression and health care utilisation, as well as more frequent HbA1c measurements than did the other two classes. Class three represented 37.4% of the study population.

**Figure 4:**
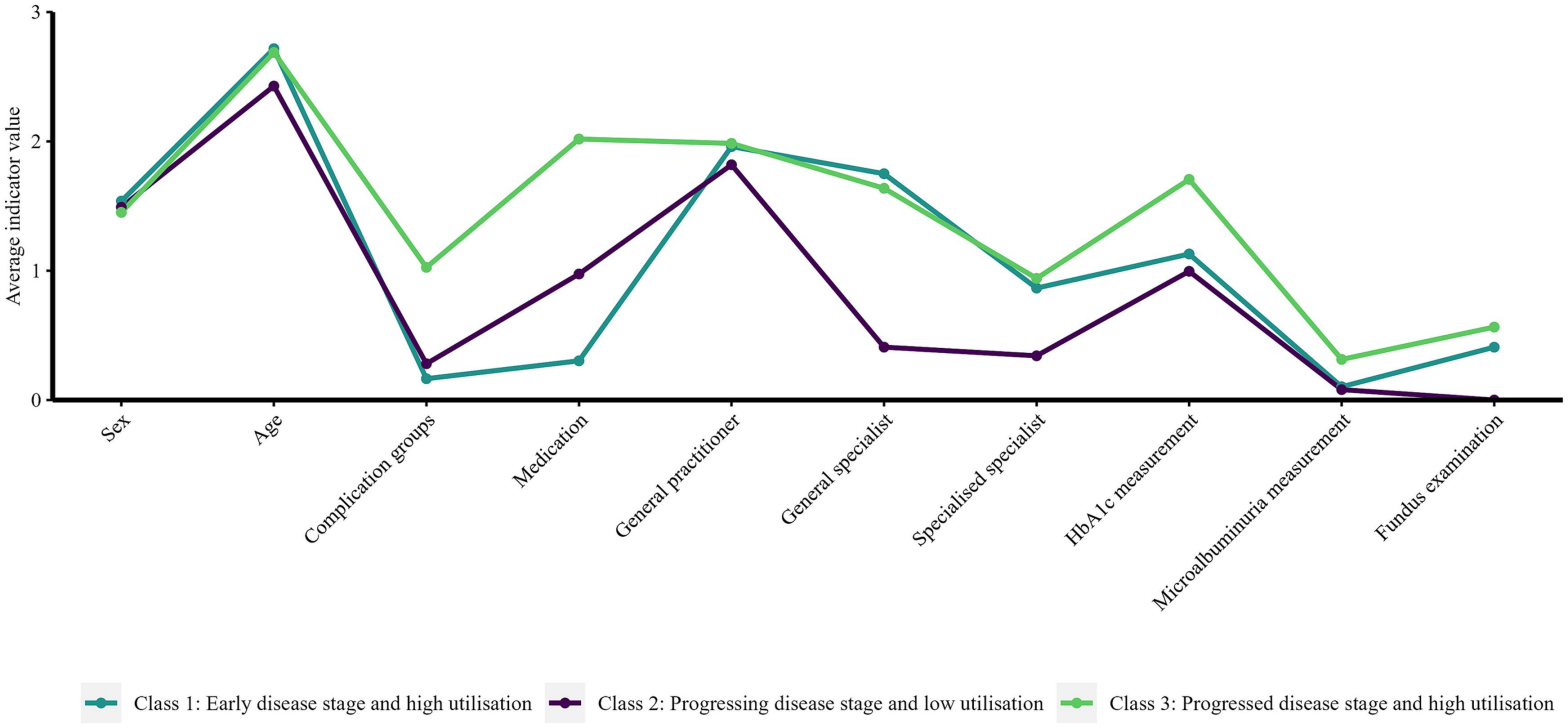
Average response values per indicator variable per class identified with the latent class analysis.

Figure 4 illustrates the average response values per indicator and class and visualises the latent class patterns. It should be noted that classes with a high average value for one variable, for example age, may also include young people and vice versa. The data points per indicator and class should not be interpreted in isolation; instead, the different graph patterns can be compared.

Although the ICD-GM-code for T2DM (E11.-) remained among the three most frequently coded ED diagnoses and main hospital diagnoses in classes two and three, it was no longer among the top ten in class one. In all three classes, congestive heart failure and cerebral infarction were the two most frequent main hospital diagnoses (Appendix 10).

In the last step, patient characteristics and the occurrence of recurrent ED visits and inpatient admissions per class were analysed (Table 7). Overall, class three had the highest comorbidity scores, with a correspondingly high proportion of recurrent ED visits and inpatient admissions. Class one had slightly higher CCI scores than did class two, which is congruent with higher health care utilisation and a higher average age in this class. However, inpatient admissions occurred more frequently, and recurrent ED visits occurred slightly more frequently in class two than in class one.

**Table 7:** Patient characteristics and occurrence of recurrent emergency department visits and inpatient admission per class identified by the latent class analysis.

Patient characteristics	Class 1 *n* = 14,797 in % (*n*)	Class 2 *n* = 10,579 in % (*n*)	Class 3 *n* = 15,185 in % (*n*)
Age in years			
20–55	9.5 (1,401)	21.8 (2,307)	6.4 (966)
56–70	24.3 (3,595)	29.1 (3,075)	27.8 (4,219)
71–85	51.4 (7,605)	33.6 (3,556)	56.6 (8,596)
≥ 86	14.9 (2,196)	15.5 (1,641)	9.2 (1,404)
Median (IQR)	76 (66, 82)	70 (58, 81)	75 (67, 81)
Sex			
Male	46.2 (6,836)	50.9 (5,380)	55.0 (8,347)
Female	53.8 (7,961)	49.1 (5,199)	45.0 (6,838)
Charlson comorbidity index^1^			
CCI Score 0	0.5 (70)	2.9 (309)	0
CCI Score 1–2	31.5 (4,655)	38.5 (4,072)	10.8 (1,642)
CCI Score 3–4	31.3 (4,634)	28.8 (3,048)	27.5 (4,170)
CCI Score ≥ 5	36.8 (5,438)	29.8 (3,148)	61.7 (9,373)
No outpatient diagnoses documented	0	<0.1 (2)	0
Median (IQR)	4 (2, 5)	3 (2, 5)	5 (4, 7)
Presence of DM-related complications^2^			
0 complication groups	83.2 (12,309)	72.3 (7,644)	7.3 (1,102)
1–2 complication groups	16.8 (2,488)	27.4 (2,901)	82.9 (12,587)
≥ 3 complications groups	0	0.3 (32)	9.9 (1,496)
No outpatient diagnoses documented	0	<0.1 (2)	0
Recurrent ED visits			
No	77.3 (11,435)	76.7 (8,116)	74.4 (11,293)
Yes	22.7 (3,362)	23.3 (2,463)	25.6 (3,892)
Inpatient admission			
No	39.2 (5,795)	33.6 (3,556)	31.7 (4,820)
Yes	60.8 (9,002)	66.4 (7,023)	68.3 (10,365)

CCI, Charlson comorbidity index, ED, Emergency department, IQR, Interquartile range; DM, Diabetes mellitus. ^1^According to Appendix 4, based on (44, 60); calculation basis: outpatient data one year prior to ED visit. ^2^According to Appendix 2; based on (47); calculation basis: outpatient data one year prior to ED visit.

Logistic regression revealed increasing odds of inpatient admissions with increasing average disease stage of T2DM per class (Table 8). The effect can already be seen between classes one and two, although class one had higher values in general comorbidity than class two. Differences were particularly visible in the age-adjusted model, in which the chance of inpatient admission in class two was even higher than that in class three.

**Table 8:** Logistic regression comparing occurrence of recurrent emergency department visits and inpatient admissions per class.

Compared outcome	Univariate regression		Age-adjusted regression	
	OR	[CI]	OR	[CI]
Recurrent ED visits				
Class 1: Early disease stage and high utilisation	1		1	
Class 2: Progressive disease stage and low utilisation	1.03	[0.97; 1.10]	**1.07**	[1.01; 1.13]
Class 3: Progressed disease stage and high utilisation	**1.17**	[1.11; 1.24]	**1.18**	[1.12; 1.24]
Inpatient admission				
Class 1: Early disease stage and high utilisation	1		1	
Class 2: Progressive disease stage and low utilisation	**1.27**	[1.21; 1.34]	**1.48**	[1.40; 1.56]
Class 3: Progressed disease stage and high utilisation	**1.38**	[1.32; 1.45]	**1.41**	[1.34; 1.48]

CI: 95%, Confidence interval; OR, Odds ratio; ED, Emergency department. Letters in bold indicate *p* < 0.05.

## 4 Discussion

### 4.1 Type II diabetes mellitus patients attending emergency departments

This study was the first to assess the diagnoses of T2DM patients in EDs in Germany using cross-sectoral claims data. With a median age of 75 years, the T2DM study population was older than the underlying ED cohort (25). As the dataset comprised individuals who had visited the ED at least once, it can be assumed that the individuals in the study population were older and at a more advanced stage of the disease compared to all T2DM patients in the general population (47). Additionally, it was already known that the application of the M2Q criterion particularly excludes younger individuals in the early stages of disease (28).

GP utilisation in the year prior to the ED visit was high, 98% of the T2DM patients had contact with a GP at least once in the first two quarters prior to the ED visit. However, for 17.8% of patients, no HbA1c measurement was billed in the year prior to the ED visit. This percentage was considerably higher than the proportion of 4.3% of T2DM patients aged 45 years and older without HbA1c measurements in the last twelve months reported by the National Diabetes Surveillance in Germany for 2021 (48, 49). Similarly, only one third (33.0%) of patients in the study population had an ophthalmological fundus examination in the year before the ED visit, whereas the proportion of T2DM patients with an eye examination in the last 12 months was 64.8%, as reported by the National Diabetes Surveillance in Germany for T2DM patients aged 45 years and older in 2021 (48). When comparing the data, it should be noted that the Diabetes Surveillance data are based on self-report survey data, not claims data (48). In addition, temporal changes between 2016 and 2021 may have contributed to the differences, although there were indications that health care utilisation for specialised care of T2DM patients decreased rather than increased in 2020 due to the starting COVID-19 pandemic (50, 51). Furthermore, the German guideline for DM treatment was adapted in 2015, as part of which the recommended period for eye examinations for people without known risk for retinopathy was adjusted from annually to biennial. This may have contributed to a reduced number of check-ups in the period under review in this study (52). Overall, 84.3% of patients in the study population did not have a microalbuminuria measurement billed in the year prior to the ED visit, but billing codes for creatinine measurement were documented for 87.2%. Similar results on creatinine measurement were reported for patients participating in the DMP for T2DM from the German federal state of North Rhine-Westphalia, ranging from 85.6 to 95.6% between 2014 and 2016 (53).

The proportion of ED visits for T2DM patients admitted as inpatients (59.0%) was notably higher than the proportion of patient visits admitted as inpatients (42.8%) in the underlying ED cohort, which is consistent with previous research (12, 25). Patients with at least one inpatient admission were on average at a more advanced stage of DM with more comorbidities than patients who were treated exclusively as outpatients. Inpatient mortality (6.6%) was about one third higher in the study population than in the underlying cohort (4.3%) (25). T2DM was the second most frequent coded ED diagnosis, as well as the third most frequent main hospital diagnosis in cases of subsequent inpatient admission. In addition, there are several diseases coded under the most frequent ED diagnoses or main hospital diagnoses that can be promoted by DM or similar risk factors as DM, such as hypertension, kidney diseases and cardiovascular diseases. Particularly striking is the high frequency of cardiovascular diagnoses, which emphasises the need for guideline-compliant check-ups for secondary diseases and consistent glycaemic control to avoid microvascular damage (37). Due to the advanced age and high comorbidity in the study population, it should be noted that presumably not all cases defined as having PAH by diagnosis were preventable in the outpatient sector, as health status as well as lifestyle-related factors not represented in the dataset are known as confounders of the relationship between access to primary care and PAH (54).

### 4.2 Segmentation of T2DM patients prior to an ED visit

Using data-driven segmentation methods, three classes differing in terms of DM-disease progression and health care utilisation were identified. It should be noted, that the labels given to the classes describe only average characteristics of the group, not on every individual person in the respective group (*“naming fallacy,”* (31)). For example, there might be few individuals in class one, *“early disease stage and high utilisation,”* with a progressed stage of T2DM, who were assigned to that class due to other characteristics like age or health care utilisation. Furthermore, the assignment of individuals to the classes was based on the information documented in the dataset. It is possible that some individuals in class one were already at a progressed stage of T2DM prior to their ED visit, despite having no visible symptoms or documented diagnoses. The LCA revealed that the variables “number of quarters with contact with a general specialist,” “medication” and “complications” differed across the identified classes (Figure 3; Appendix 8). This indicates that both disease progression and specialist utilisation are important for interpreting the patient groups. These findings highlight the potential for developing targeted care strategies in integrated care settings.

On average, the indicators used to measure the progression of T2DM showed an early stage of disease in the first class. However, since the CCI scores indicated a pronounced level of comorbidity, it can be assumed that this class would benefit from preventive measures targeting risk factors promoting a range of non-communicable diseases. In contrast, class two showed on average an advanced disease stage of T2DM and at the same time the lowest CCI values. Beyond the prevention of primary risk factors, the focus for this group should be on secondary prevention of T2DM in the form of reducing disease progression and associated complications. In both class one and class two, the recommended frequency of quarterly HbA1c measurements was met by only approximately one third of patients in the respective classes. This indicates a high potential for improving process quality in the outpatient care sector in both classes (48). For class three, a high overall morbidity regarding T2DM as well as other diseases could be shown. For this group, the focus should be on ensuring continuity of care and thus on avoiding disruptions of care. Instruments of managed care, e.g., DMPs, GP-centred gatekeeping or case management by home-nursing units, offer a promising approach to both reducing emergency cases and ensuring better cross-sectoral continuity of care in cases of unavoidable ED visits or inpatient admissions (55). For clinicians in the outpatient setting, access to information on patients’ participation in regular check-ups, existing specialist care, and any complications related to T2DM is essential. This information enables the clinician to more accurately assess their patients’ condition and adapt their treatment accordingly. Guideline-based treatment has the potential to prevent the disease from progressing and thus reduce the risk for emergency treatment.

Regarding PAH, the ten most frequently coded main hospital diagnoses per class suggest that in classes two and three PAH occurred more often directly due to T2DM, among other causes, while T2DM had more of an indirect effect on the occurrence of PAH in class one. Beyond that, it could be confirmed that the risk of inpatient admission increased in classes with an average advanced stage of T2DM (12). In comparison, class two with a less pronounced CCI but more advanced T2DM even had a higher risk for inpatient admissions than class one with a slightly more pronounced average CCI but less pronounced T2DM. The higher chance of inpatient admission in class two than in class three was more visible in the age-adjusted model than in the crude model, however, this finding must be interpreted with caution, as age was already included as an indicator variable in class partitioning. Further research is necessary to specify the health care needs of the identified classes in detail to plan and evaluate strategies that can reduce the proportion of PAH patient cases.

From a methodological perspective, the combination of values assessing model fit and values assessing cluster separation ensured interpretability (40). The graphs running parallel in some sections in Figure 4 suggest that the so-called salsa effect may have occurred in the analysis. This effect describes the phenomenon in which different patterns were not identified, but rather the same pattern in varying degrees of severity [*“mild, medium and hot salsa,”* (30)]. This phenomenon does not necessarily represent a methodological error but must be considered in interpreting the classes (31). The occurrence of the salsa effect was accepted for this study since interactions between disease stages and health care utilisation contribute to the understanding of disease trajectories and health care needs. The slight salsa effect, particularly between the variables of health care utilisation and process quality, can be explained by the fact that no examination would have taken place without prior contact with the responsible physician and that two variables indicating disease progression were included in the model for class partitioning. It can be assumed that the effect would have been smaller if other indicator variables had been included that reflect not only the utilisation of services provided by physicians but also of other professions, such as outpatient home nursing, podiatry, nutrition assistants or specialised outpatient wound care. In addition to other health care levels, further studies should consider class differences in terms of socioeconomic status, lifestyle-related factors, physician density or living in a deprived environment, which are already known to be associated with the utilisation of health care services and the occurrence of PAH (54, 56).

### 4.3 Potential limitations

The dataset offered the opportunity to map cross-sectoral health care utilisation via the ED as an interface between the inpatient and outpatient sectors. However, several limitations have to be mentioned. First, providers in the outpatient sector show different coding practices due to low-standardised coding guidelines (29, 57). Second, due to the structure of the dataset and the quality of coding diagnoses in EDs in Germany, it was not clear whether DM, if coded as a diagnosis in the ED, was the main cause for the ED visit or whether it was coded as a concomitant disease. The lack of differentiation between principal and secondary diagnoses in the ED dataset was one of the main limitations of the dataset and highlights well-known challenges of data quality in the German outpatient sector (57). This limitation does not apply for the main hospital diagnoses, as only one main hospital diagnosis was documented per inpatient case. Since T2DM and several diseases that are secondary to T2DM were among the top ten inpatient diagnoses, we assume that in many cases DM contributed at least in part to the need for the ED visit.

Third, the lack of complete data on DMP participation in the underlying dataset represented a further limitation and hinders the evaluation of an already well-implemented programme (1, 58, 59). If insights had been available to determine whether a large proportion of T2DM patients were not enrolled in a DMP, this would have clearly highlighted the need to promote participation in such programmes. Fourth, the lack of data on drug prescriptions from one of the participating associations of SHI physicians may have led to the exclusion of T2DM patients from the study population due to the applied criteria for diagnosis validation. Fifth, in addition, the outpatient claims data do not contain any quantitative clinical data, for example on the level of measured HbA1c or other laboratory parameters. Sixth, no lifestyle-related information, for example on physical activity, dietary behaviour or social support as well as socioeconomic status was available to evaluate their association with T2DM healthcare provision in Germany. Socioeconomic and lifestyle-related factors as well as HbA1c levels have already been shown to be relevant factors when segmenting T2DM patients into subgroups (17). This information could have contributed to practical recommendations for improving the quality of outpatient care – such as not only regularly measuring HbA1c, but also adjusting treatment regimens when values fall outside the target range. Further research is necessary to develop data driven recommendations.

Seventh, since not all associations of SHI physicians in Germany transmitted outpatient data, the ED patients may have had contact with a physician billed through one of the associations of SHI physicians not represented in the dataset. Therefore, it was not possible to distinguish whether individuals who did not appear in the outpatient data prior to the ED visit have never had contact with a physician or have had a physician contact that was billed via an association of SHI physicians not included in the study and thus not transmitted to the study site. In addition, no information is known about ED visits or inpatient admissions before 2016 or visits to EDs that are not part of the INDEED study or the utilisation of services that are not covered by the SHI, i.e., have to be paid out of pocket. The dataset therefore did not allow us to draw conclusions about total lack of access to health care prior to the ED visit or first-time diagnoses in the ED. Identification of first-time diagnoses of DM in the ED was additionally prevented by the application of the M2Q criterion for diagnosis validation, as individuals without at least one outpatient diagnosis or DM-medication use prior to the ED visit or DM as the main hospital diagnosis were excluded from the study population.

Eighth, it should be noted that the available data and period under consideration did not allow us to draw conclusions on any causal inference between health care utilisation and disease stage. For example, no conclusions can be drawn as to whether low health care utilisation occurred due to a lack of need in the case of low morbidity, or whether low morbidity occurred due to the lack of documented diagnoses due to low utilisation.

## 5 Conclusion

The results of the indicators measuring the frequency of medical examinations recommended by guidelines suggest the potential for improving process quality in the outpatient sector to prevent ED utilisation. Our study showed that segmenting the T2DM population according to their health care utilisation contributes to a better understanding of cross-sectoral patterns of care. We identified three latent classes of T2DM characterised as *“early disease stage and high utilisation*” (36.5% of the study population), “*progressing disease stage and low utilisation”* (26.1%) and *“progressed disease stage and high utilisation”* (37.4%). This can contribute to plan and establish health care measures aimed at reducing PAH and to improve health care continuity. Nevertheless, further research is necessary to identify and evaluate respective interventions per identified class. In addition, further research should investigate the role of non-physician health care providers as well as socioeconomic and lifestyle-related factors in cross-sectoral care patterns.

## Data Availability

The data analyzed in this study is subject to the following licenses/restrictions: due to the high sensitivity of the data in the project, it is not possible to make those available to the public. In the central data management of the project the data are already pseudonymised twice. Data are made available exclusively to the analyzing partners of the consortium in anonymised form and only with variables that are matched to the research question. Requests to access these datasets should be directed to antje.fischer-rosinsky@charite.de.
