# Supplementary material for: Data-driven segmentation of type 2 diabetes mellitus patients: an observational study on health care utilisation prior to an emergency department visit in Germany
Source: Front Med (Lausanne). 2025 May 16;12:1509220. doi: 10.3389/fmed.2025.1509220 (PMC12122753; doi:10.3389/fmed.2025.1509220)
Supplement: Supplementary file 1 [file Data_Sheet_1.docx]

Supplementary Material

Data-driven segmentation of type 2 diabetes mellitus patients: Health care utilisation prior to an emergency department visit in Germany

Mirjam Rupprecht^1^*, Alessandro Campione^2^, Yves Noel W^3^, Antje Fischer-Rosinský^3^, Anna Slagman^3^, Dorothee Riedlinger^3^, Martin Möckel^3^, Thomas Keil^4,5, 6^, Lukas Reitzle^7†^, Cornelia Henschke^2,8†^

^1^Department for Infectious Disease Epidemiology, Robert Koch Institute, Berlin, Germany

^2^Department of Health Care Management, Berlin Centre for Health Economics Research, Technische Universität Berlin, Berlin, Germany

^3^Emergency and Acute Medicine (CVK, CCM), Charité - Universitätsmedizin Berlin, Berlin, Germany

^4^Institute of Social Medicine, Epidemiology and Health Economics, Berlin, Germany; Charité - Universitätsmedizin Berlin;

^5^Institute of Clinical Epidemiology and Biometry, University of Würzburg, Würzburg, Germany;

^6^State Institute of Health I, Bavarian Health and Food Safety Authority, Erlangen, Germany

^7^Department of Epidemiology and Health Monitoring, Robert Koch Institute, Berlin, Germany

^8^Institute of General Practice and Interprofessional Care, Faculty of Medicine, Eberhard Karls Universität Tübingen, Germany

***Correspondence:**Mirjam Rupprecht
[RupprechtM@rki.de](mailto:RupprechtM@rki.de)

# Appendix 1: Algorithm for the detection of type 2 diabetes mellitus

In case of discrepancies between the recommendations of RKI and IQWiG, cases were excluded to reliably narrow down the study population.

| **Description** | **Criteria** | | | | | | | | | **Incl.** | **Excl.** |
| --- | --- | --- | --- | --- | --- | --- | --- | --- | --- | --- | --- |
|  | **Diagnosis** | | | | | **Medication** | | **Participation in DMP** | |  |  |
|  | **E10** | **E11** | **E12** | **E13** | **E14** | **Insulin** | **OAD** | **Type 1** | **Type 2** |  |  |
| Only E10 | 1 | 0 | 0 | 0 | 0 | * | * | * | * | 0 | 1 |
| Only E11 | 0 | 1 | 0 | 0 | 0 | * | * | * | * | 1 | 0 |
| Only E12 | 0 | 0 | 1 | 0 | 0 | * | * | * | * | 1 | 0 |
| Only E13 | 0 | 0 | 0 | 1 | 0 | * | * | * | * | 0 | 1 |
| Only E14 | 0 | 0 | 0 | 0 | 1 | 0 | 0 | 0 | 0 | 1 | 0 |
|  | 0 | 0 | 0 | 0 | 1 | 0 | 0 | 0 | 1 | 1 | 0 |
|  | 0 | 0 | 0 | 0 | 1 | 0 | 0 | 1 | 1 | 0 | 1 |
|  | 0 | 0 | 0 | 0 | 1 | 1 | 1 | * | * | 1 | 0 |
|  | 0 | 0 | 0 | 0 | 1 | 0 | 1 | * | * | 1 | 0 |
|  | 0 | 0 | 0 | 0 | 1 | 1 | 0 | 0 | 0 | 0 | 1 |
|  | 0 | 0 | 0 | 0 | 1 | 1 | 0 | 0 | 1 | 1 | 0 |
|  | 0 | 0 | 0 | 0 | 1 | 1 | 0 | 1 | 0 | 0 | 1 |
| E10 and E11 | 1 | 1 | * | * | * | 0 | 0 | * | * | 1 | 0 |
|  | 1 | 1 | * | * | * | 1 | 1 | * | * | 1 | 0 |
|  | 1 | 1 | * | * | * | 0 | 1 | * | * | 1 | 0 |
|  | 1 | 1 | * | * | * | 1 | 0 | 0 | 0 | 0 | 1 |
|  | 1 | 1 | * | * | * | 1 | 0 | 0 | 1 | 1 | 0 |
|  | 1 | 1 | * | * | * | 1 | 0 | 1 | 0 | 0 | 1 |
|  | 1 | 1 | * | * | * | 1 | 0 | 1 | 1 | 0 | 1 |
| E10 and E12 | 1 | 0 | 1 | * | * | 0 | 0 | * | * | 1 | 0 |
|  | 1 | 0 | 1 | * | * | 1 | 1 | * | * | 1 | 0 |
|  | 1 | 0 | 1 | * | * | 0 | 1 | * | * | 1 | 0 |
|  | 1 | 0 | 1 | * | * | 1 | 0 | 0 | 0 | 0 | 1 |
|  | 1 | 0 | 1 | * | * | 1 | 0 | 0 | 1 | 1 | 0 |
|  | 1 | 0 | 1 | * | * | 1 | 0 | 1 | 0 | 0 | 1 |
| E10 and E13 | 1 | 0 | 0 | 1 | * | 1 | 0 | * | * | 0 | 1 |
|  | 1 | 0 | 0 | 1 | * | 0 | 1 | 0 | 0 | 0 | 1 |
|  | 1 | 0 | 0 | 1 | * | 1 | 1 | 0 | 0 | 0 | 1 |
|  | 1 | 0 | 0 | 1 | * | 0 | 0 | 0 | 0 | 0 | 1 |
| E10 and E14 | 1 | 0 | 0 | 0 | 1 | 1 | 1 | 0 | 0 | 0 | 1 |
|  | 1 | 0 | 0 | 0 | 1 | 1 | 1 | 1 | 0 | 0 | 1 |
|  | 1 | 0 | 0 | 0 | 1 | 1 | 1 | 0 | 1 | 1 | 0 |
|  | 1 | 0 | 0 | 0 | 1 | 0 | 0 | 0 | 0 | 0 | 1 |
|  | 1 | 0 | 0 | 0 | 1 | 0 | 0 | 0 | 1 | 1 | 0 |
|  | 1 | 0 | 0 | 0 | 1 | 1 | 0 | * | * | 0 | 1 |
|  | 1 | 0 | 0 | 0 | 1 | 0 | 1 | 0 | 0 | 0 | 1 |
|  | 1 | 0 | 0 | 0 | 1 | 0 | 1 | 0 | 1 | 1 | 0 |
| E11 and E12 | 0 | 1 | 1 | * | * | * | * | * | * | 1 | 0 |
| E11 and E13 | 0 | 1 | * | 1 | * | * | * | * | * | 1 | 0 |
| E11 and E14 | 0 | 1 | * | * | 1 | * | * | * | * | 1 | 0 |
| E12 and E13 | 0 | 0 | 1 | 1 | * | * | * | * | * | 1 | 0 |
| E12 and E14 | 0 | 0 | 1 | * | 1 | * | * | * | * | 1 | 0 |
| E13 and E14 | 0 | 0 | 0 | 1 | 1 | * | * | * | * | 0 | 1 |

*Abbr: Incl: Inclusion, DMP: Disease management program; Excl.: Exclusion; OAD: Oral antidiabetic drug*

*0: does not exist; 1: exists, *not defined, can exist or not exist*

# Appendix 2: Overview of ICD-codes used for definition of diabetes-related complication groups

| **Complication group** | **ICD-10-codes** | **Description** |
| --- | --- | --- |
| Metabolic system | E[10-14].0  E[10-14].1 | DM with coma  DM with ketoacidosis |
| Kidney | E[10-14].2  N08.3 | DM with renal complications  Glomerular diseases in diabetes mellitus |
| Eye | E[10-14].3  H36.0  H28.0 | DM with ophthalmic complications  Diabetic retinopathy  Diabetic cataract |
| Neurovascular system | E[10-14].4  G63.2  G59.0  E[10-14].5  I79.2  E[10-14].73  E[10-14].74  M14.2 | DM with neurological complications  Diabetic polyneuropathy  Diabetic mononeuropathy  DM with peripheral circulatory complications  Peripheral diabetic angiopathy  DM with diabetic foot syndrome, controlled  DM with diabetic foot syndrome, uncontrolled  Diabetic arthropathy |
| Multiple complications | E[10-14].71  E[10-14].72 | DM with other multiple complications, controlled  DM with other multiple complications, uncontrolled |

The ICD codes correspond to the ICD-10-GM version 2016 [19]. The "multiple complications"-class was only counted if there were no codes existent for any of the other groups. In this case, two points were awarded for the existence of a code in the "multiple complications" group. One point was awarded per one of the other complication groups if one of the ICD-codes listed existed.

# Appendix 3: Overview of included DM-relevant disciplines per physician group

| **Physician Group** | **German professional group designation of included disciplines** |
| --- | --- |
| General practitioner | General medicine  Physician without further specialist training  Internist specialised on general medicine |
| General specialist | Ophthalmology  Surgery  Vascular surgery  Visceral surgery  Orthopaedics and trauma surgery  Plastic surgery  Dermatology  Neurologist/ Neurology and Psychiatry  Neurology  Physical and rehabilitative medicine  Psychiatry/ Psychiatry and Psychotherapy  Psychosomatic medicine  Psychotherapeutic physician  Urology  Psychological psychotherapy |
| Specialised Specialists | Specialised Internists  Angiology  Endocrinology/ Diabetology  Gastroenterology  Cardiology  Nephrology  Geriatrics  Radiology  Neuroradiology |

# Appendix 4: Coding of Charlson comorbidity index

CCI was coded according to the R algorithm provided by Quan et al. [46]. The following diagnoses were considered by the algorithm:

| **Comorbidities** | **ICD-10** |
| --- | --- |
| Myocardial infarction | I21.x, I22.x, I25.2 |
| Congestive heart failure | I09.9, I11.0, I13.0, I13.2, I25.5, I42.0, I42.5–I42.9, I43.x, I50.x, P29.0 |
| Peripheral vascular disease | I70.x, I71.x, I73.1, I73.8, I73.9, I77.1, I79.0, I79.2, K55.1, K55.8, K55.9, Z95.8, Z95.9 |
| Cerebrovascular disease | G45.x, G46.x, H34.0, I60.x–I69.x |
| Dementia | F00.x–F03.x, F05.1, G30.x, G31.1 |
| Chronic pulmonary disease | I27.8, I27.9, J40.x–J47.x, J60.x–J67.x, J68.4, J70.1, J70.3 |
| Rheumatic disease | M05.x, M06.x, M31.5, M32.x–M34.x, M35.1, M35.3, M36.0 |
| Peptic ulcer disease | K25.x–K28.x |
| Mild liver disease | B18.x, K70.0–K70.3, K70.9, K71.3–K71.5, K71.7, K73.x, K74.x, K76.0, K76.2–K76.4, K76.8, K76.9, Z94.4 |
| Diabetes without chronic complication | E10.0, E10.1, E10.6, E10.8, E10.9, E11.0, E11.1, E11.6, E11.8, E11.9, E12.0, E12.1, E12.6, E12.8, E12.9, E13.0, E13.1, E13.6, E13.8, E13.9, E14.0, E14.1, E14.6, E14.8, E14.9 |
| Diabetes with chronic complication | E10.2–E10.5, E10.7, E11.2–E11.5, E11.7, E12.2–E12.5, E12.7, E13.2– E13.5, E13.7, E14.2–E14.5, E14.7 |
| Hemiplegia or paraplegia | G04.1, G11.4, G80.1, G80.2, G81.x, G82.x, G83.0–G83.4, G83.9 |
| Renal disease | I12.0, I13.1, N03.2–N03.7, N05.2– N05.7, N18.x, N19.x, N25.0, Z49.0–Z49.2, Z94.0, Z99.2 |
| Any malignancy, including lymphoma  and leukaemia, except malignant neoplasm of skin | C00.x–C26.x, C30.x–C34.x, C37.x–C41.x, C43.x, C45.x–C58.x, C60.x–C76.x, C81.x–C85.x, C88.x, C90.x–C97.x |
| Moderate or severe liver disease | I85.0, I85.9, I86.4, I98.2, K70.4, K71.1, K72.1, K72.9, K76.5, K76.6, K76.7 |
| Metastatic solid tumour | C77.x–C80.x |
| AIDS/HIV | B20.x–B22.x, B24.x |

A hierarchy of comorbidities was applied. This means that if a comorbidity was present in a patient with different degrees of severity, only the more severe form was considered. Only diagnoses with the addition "confirmed" or "status after" were considered. No further diagnosis validation such as the M2Q criterion was applied. Weighting of diseases was conducted according to the original recommendation by Charlson et al. [49].

# Appendix 5: Detailed overview of E11.- diagnoses documented as emergency department diagnosis (EDD) or main hospital diagnosis (MHD)

| **ICD-Code** | | **ICD-Code description:**  **T2DM** | **EDDs**  **n = 1,398** | **MHDs**  **n = 1,043** |
| --- | --- | --- | --- | --- |
| E11 | Without further description (Coding error) | | 0.1 (2) | 0 |
| E11.0 | With coma | | 1.6 (22) | 3.9 (41) |
| E11.1 | With ketoacidosis | | 0.5 (7) | 1.2 (12) |
| E11.2 | With renal complications  Controlled  Uncontrolled | | 3.1 (44)  2.1 (29) | 0.8 (8)  2.0 (21) |
| E11.3 | With ophthalmic complications  Controlled  Uncontrolled | | 1.1 (16)  0.6 (8) | 0.9 (9)  0 |
| E11.4 | With neurological complications  Controlled  Uncontrolled | | 3.7 (52)  1.2 (17) | 4.7 (49)  2.9 (30) |
| E11.5 | With peripheral circulatory complications  Controlled  Uncontrolled | | 4.5 (63)  0.3 (4) | 1.0 (10)  1.0 (10) |
| E11.6 | With other specified complications  Controlled  Uncontrolled | | 1.6 (23)  1.4 (20) | 2.4 (25)  17.4 (181) |
| E11.7 | With multiple complications  With other multiple complications, controlled  With other multiple complications, uncontrolled  With diabetic foot syndrome, controlled  With diabetic foot syndrome, uncontrolled | | 4.6 (65)  3.5 (49)  2.4 (33)  1.1 (16) | 1.2 (13)  9.7 (101)  17.2 (179)  7.4 (77) |
| E11.8 | With unspecified complications  Controlled  Uncontrolled | | 0.5 (7)  1.9 (27) | 0  0 |
| E11.9 | Without complications  Controlled  Uncontrolled | | 46.8 (654)  17.2 (240) | 1.1 (11)  25.5 (266) |
|  | *Total share of diagnoses described as uncontrolled* | | *31.4 (438)* | *71.0 (739)* |

The ICD-codes E11.0 (T2DM with coma) and E11.1 (T2DM with ketoacidosis) describe uncontrolled conditions by definition. With an average of 9.9 documented diagnoses per ED visit, one of the 13 EDs was significantly above the average (2.4 diagnoses/ ED visit) and was excluded from the analysis of the top ten most frequently coded EDDs.

# Appendix 6: Patterns of missing data in indicator variables used for LCA





*s. extra file Appendix6_MissingValuePatern for .tiff-file*

# Appendix 7: Correlation matrix of indicator variables used for LCA

|  | **Sex** | **Age** | **Compl** | **Med** | **GP** | **GenS** | **SpecS** | **HbA1c** | **Crea** | **MU** | **FE** |
| --- | --- | --- | --- | --- | --- | --- | --- | --- | --- | --- | --- |
| **Sex** | 1.00 | -0.13 | 0.04 | 0.06 | -0.04 | 0.01 | 0.04 | 0.02 | 0.03 | 0.01 | <0.01 |
| **Age** | -0.13 | 1.00 | 0.10 | -0.05 | 0.12 | 0.12 | -0.04 | -0.01 | 0.03 | -0.04 | 0.08 |
| **Compl** | 0.04 | 0.10 | 1.00 | 0.49 | 0.11 | 0.13 | 0.12 | 0.28 | 0.16 | 0.14 | 0.19 |
| **Med** | 0.06 | -0.05 | 0.49 | 1.00 | 0.07 | <0.01 | 0.02 | 0.33 | 0.16 | 0.14 | 0.13 |
| **GP** | -0.04 | 0.12 | 0.11 | 0.07 | 1.00 | 0.14 | 0.05 | 0.19 | 0.18 | 0.07 | 0.08 |
| **GenS** | 0.01 | 0.12 | 0.13 | <0.01 | 0.14 | 1.00 | 0.22 | 0.11 | 0.14 | 0.06 | 0.32 |
| **SpecS** | 0.04 | -0.04 | 0.12 | 0.02 | 0.05 | 0.22 | 1.00 | 0.16 | 0.22 | 0.07 | 0.15 |
| **HbA1c** | 0.02 | -0.01 | 0.28 | 0.33 | 0.19 | 0.11 | 0.16 | 1.00 | **0.63** | 0.24 | 0.19 |
| **Crea** | 0.03 | 0.03 | 0.16 | 0.16 | 0.18 | 0.14 | 0.22 | **0.63** | 1.00 | 0.15 | 0.17 |
| **MU** | 0.01 | -0.04 | 0.14 | 0.14 | 0.07 | 0.06 | 0.07 | 0.24 | 0.15 | 1.00 | 0.09 |
| **FE** | <0.01 | 0.08 | 0.19 | 0.13 | 0.08 | 0.32 | 0.15 | 0.19 | 0.17 | 0.09 | 1.00 |

# Appendix 8: Discriminative power of indicator variables


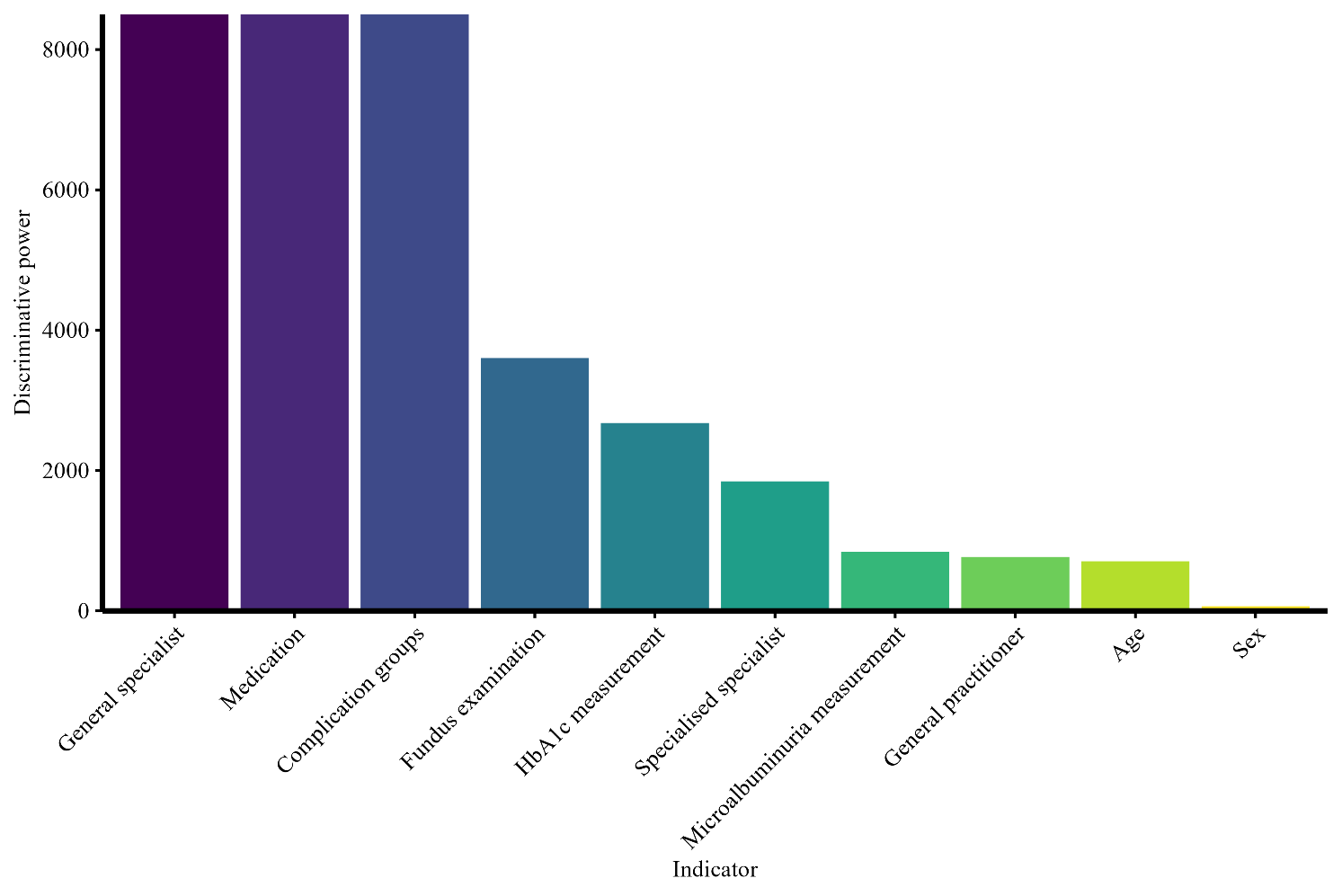


*s. extra file Appendix8_DiscriminativePower for .tiff-file*

# Appendix 9: Average latent class posterior probability per class

| **Predicted class** | **Average latent class posterior probability** |
| --- | --- |
| Class 1: Early disease stage and high utilisation | 0.80 |
| Class 2: Progressive disease stage and low utilisation | 0.86 |
| Class 3: Progressed disease stage and high utilisation | 0.89 |

# Appendix 10: Top ten most frequent ICD-10-GM-codes documented as ED diagnosis or main hospital diagnosis per class

*Top ten ED diagnoses*

|  | **Class 1, n = 23,717** | | **Class 2, n = 17,638** | | **Class 3, n = 25,841** | |
| --- | --- | --- | --- | --- | --- | --- |
| **Rank** | **Code** | **in % (n)** | **Code** | **in % (n)** | **Code** | **in % (n)** |
| **1** | I10 | 2.6 (625) | I10 | 2.5 (444) | E11 | 2.7 (709) |
| **2** | M54 | 2.1 (493) | E11 | 2.4 (432) | I10 | 2.4 (610) |
| **3** | S00 | 2.0 (465) | R10 | 1.9 (327) | I50 | 2.0 (529) |
| **4** | R10 | 1.7 (406) | I63 | 1.7 (298) | J18 | 1.9 (486) |
| **5** | S01 | 1.7 (397) | S00 | 1.7 (296) | I63 | 1.8 (477) |
| **6** | R07 | 1.6 (384) | J18 | 1.6 (277) | M54 | 1.7 (438) |
| **7** | I48 | 1.5 (363) | R07 | 1.5 (264) | I48 | 1.5 (391) |
| **8** | Z03 | 1.5 (356) | E87 | 1.5 (256) | R10 | 1.5 (379) |
| **9** | I63 | 1.4 (340) | I50 | 1.4 (254) | R06 | 1.5 (379) |
| **10** | J18 | 1.4 (340) | R06 | 1.4 (249) | S00 | 1.5 (377) |

*Top ten main hospital diagnoses*

|  | **Class 1 n = 11,038** | | **Class 2, n = 8,886** | | **Class 3, n = 13,300** | |
| --- | --- | --- | --- | --- | --- | --- |
| **Rank** | **Code** | **in % (n)** | **Code** | **in % (n)** | **Code** | **in % (n)** |
| **1** | I63 | 6.0 (660) | I63 | 6.3 (556) | I50 | 6.3 (839) |
| **2** | I50 | 4.2 (464) | I50 | 5.4 (481) | I63 | 6.1 (816) |
| **3** | S06 | 3.6 (396) | E11 | 4.1 (362) | E11 | 4.1 (542) |
| **4** | G45 | 2.5 (273) | I21 | 3.4 (299) | I21 | 3.2 (429) |
| **5** | A41 | 2.3 (250) | A41 | 3.0 (271) | A41 | 3.2 (422) |
| **6** | J18 | 2.2 (243) | S06 | 2.5 (219) | S06 | 2.9 (389) |
| **7** | I20 | 2.2 (242) | J18 | 2.2 (195) | N17 | 2.6 (342) |
| **8** | I48 | 2.1 (234) | S72 | 2.1 (186) | I20 | 2.1 (283) |
| **9** | I21 | 2.1 (229) | J44 | 2.1 (184) | J18 | 2.1 (276) |
| **10** | J44 | 1.9 (208) | N17 | 1.9 (168) | G45 | 2.0 (271) |

The ICD-codes correspond to the ICD-10-GM version 2016 [21].

*Legend*

| A41 | Other sepsis |
| --- | --- |
| E11 | Type 2 diabetes mellitus |
| E87 | Other disturbances of the water and electrolyte balance as well as the acid-base balance |
| G45 | Cerebral transient ischaemia and related syndromes |
| I10 | Essential (primary) hypertension |
| I20 | Angina pectoris |
| I21 | Acute myocardial infarction |
| I48 | Atrial fibrillation and flutter |
| I50 | Congestive heart failure |
| I63 | Cerebral infarction |
| J18 | Pneumonia, pathogen not specified |
| J44 | Other chronic obstructive pulmonary disease |
| M54 | Back pain |
| N17 | Acute renal failure |
| R06 | Respiratory disorders |
| R07 | Throat and chest pain |
| R10 | Abdominal and pelvic pain |
| S00 | Superficial injury to the head |
| S01 | Open wound to the head |
| S06 | Intracranial injury |
| S72 | Femur fracture |
| Z03 | Medical observation and assessment of suspected cases |
