# Supplementary figures and images for: Data-driven segmentation of type 2 diabetes mellitus patients: an observational study on health care utilisation prior to an emergency department visit in Germany
Source: Front Med (Lausanne). 2025 May 16;12:1509220. doi: 10.3389/fmed.2025.1509220 (PMC12122753; doi:10.3389/fmed.2025.1509220)

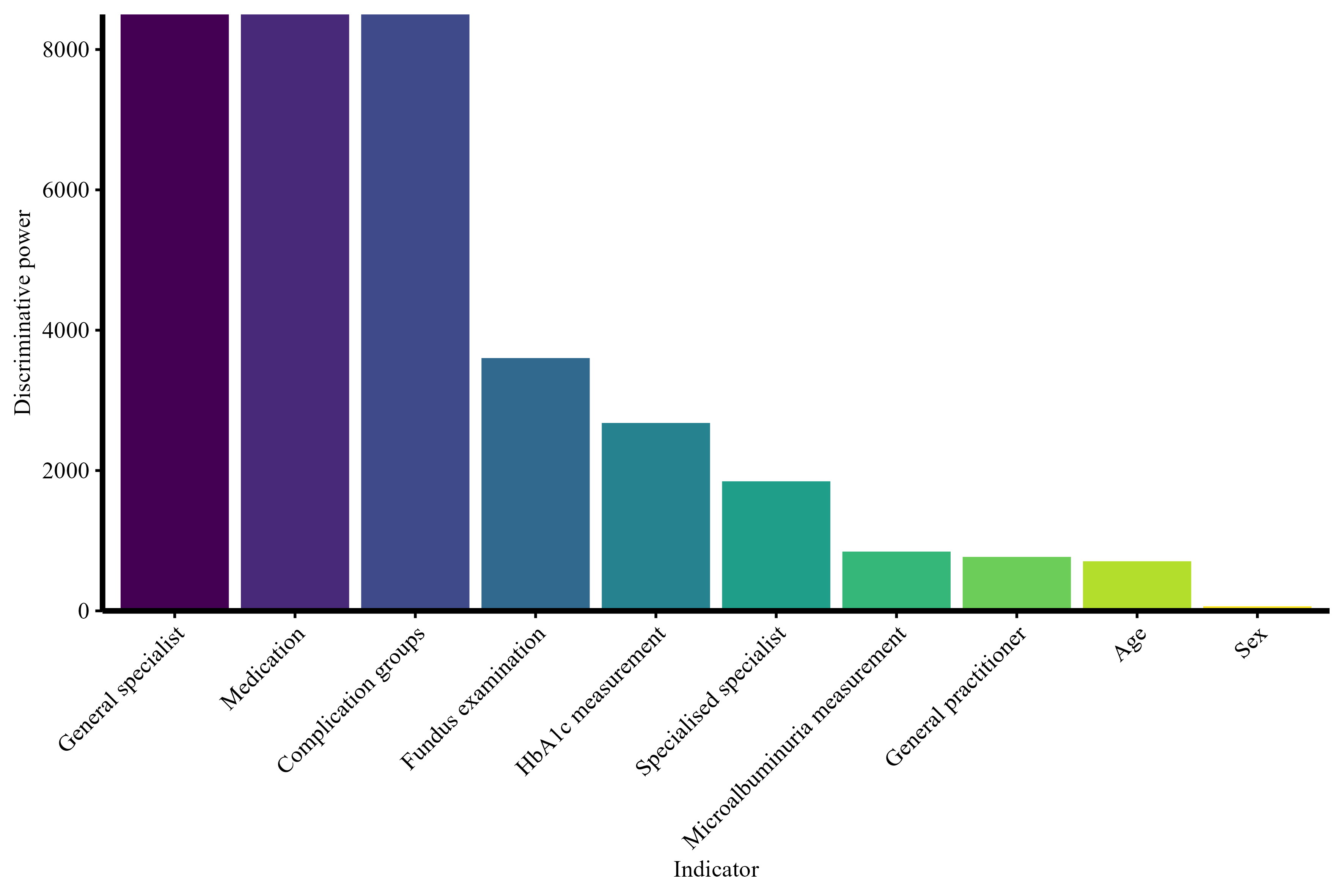

Supplement: Supplementary file 2 [file Image_1.tiff]
